# Molecular Dynamics Simulations on the Adsorbed Monolayers of N-Dodecyl Betaine at the Air–Water Interface

**DOI:** 10.3390/molecules28145580

**Published:** 2023-07-22

**Authors:** Chengfeng Zhang, Lulu Cao, Yongkang Jiang, Zhiyao Huang, Guokui Liu, Yaoyao Wei, Qiying Xia

**Affiliations:** School of Chemistry and Chemical Engineering, Linyi University, Linyi 276000, China; zcf100660@163.com (C.Z.); 19560890625@163.com (L.C.); justy838@163.com (Y.J.); yaoshuu@163.com (Z.H.); liuguokui@lyu.edu.cn (G.L.)

**Keywords:** betaine, molecular dynamics simulations, air-water interface, monolayer structure

## Abstract

Betaine is a kind of zwitterionic surfactant with both positive and negative charge groups on the polar head, showing good surface activity and aggregation behaviors. The interfacial adsorption, structures and properties of *n*-dodecyl betaine (NDB) at different surface coverages at the air–water interface are studied through molecular dynamics (MD) simulations. Interactions between the polar heads and water molecules, the distribution of water molecules around polar heads, the tilt angle of the NDB molecule, polar head and tail chain with respect to the surface normal, the conformations and lengths of the tail chain, and the interfacial thickness of the NDB monolayer are analyzed. The change of surface coverage hardly affects the locations and spatial distributions of the water molecules around the polar heads. As more NDB molecules are adsorbed at the air–water interface, the number of hydrogen bonds between polar heads and water molecules slightly decreases, while the lifetimes of hydrogen bonds become larger. With the increase in surface coverage, less gauche defects along the alkyl chain and longer NDB chain are obtained. The thickness of the NDB monolayer also increases. At large surface coverages, tilted angles of the polar head, tail chain and whole NDB molecule show little change with the increase in surface area. Surface coverages can change the tendency of polar heads and the tail chain for the surface normal.

## 1. Introduction

Betaine widely exists in animals and plants [1,2]. It is a kind of important osmotic adjustment substance. For plants, betaine contributes to enhance stress resistance, such as salt resistance and drought resistance. When the tail chain becomes longer, betaine possesses interfacial activity. These betaines with long tail chains are classified to be zwitterionic surfactants, which are important kinds of surfactants and have important applications in industry and life [3].

Due to the wide application of betaine, it has attracted much scientific attention. Liu et al. used surface tension and small angle neutral-scattering methods to study the interaction between four kinds of betaines and the lipopeptide surfactin. They found that the synergistic effect between them was related to the structure and molar ratio of betaine [4]. Hines et al. studied the aggregation structure of n-dodecyl-N,N-dimethylamino acetate amphoteric surfactants at the gas–liquid interface using the neutral reflection method, and they proposed the aggregation model of related systems at the gas–liquid interface [5]. Through surface tension and thermokinetic analysis, Erfani et al. studied the effect of cocamidopropyl betaine on the adsorption of bovine serum albumin at the gas–liquid interface. The results showed that betaine could effectively prevent the adsorption of protein at the interface [6]. With small angle neutral scaling and visibility measurements, McCoy et al. [7] studied the effect of organic additives on the micelles formed by oleyl amidopropyl betaine. When nonpolar organic additives are added, wormlike micelles could transform into microemulsions, while polar additives only affected fluid rheology, not changing micelles into microemulsions. Gao et al. [8] compared the interfacial adsorption of alkylbetaine with the one of phenyl-containing betaine, showing that betaine with a benzene ring has better interfacial activity. Hussain et al. [9] studied the effect of different ethoxylation on the properties of betaine so as to study the betaine that can be dissolved in aqueous solution containing salt.

In addition to experimental research, molecular dynamics (MD) simulation also plays an important role in studying surfactant systems [10,11,12,13,14,15]. Liu et al. used the MD simulation method to study the structural characteristics of several betaines and carboxylic acid mixtures at the n-decane–water interface. They found that polar head tends to be parallel to the interface, while the tail tends to be in the oil phase. The benzene ring on an alkyl chain would affect the distribution of the tail chain [16]. Furthermore, Cai et al. studied the microstructure characteristics of two betaines and three ionic surfactants mixtures at the n-decane–water interface by MD simulation, focusing on the changes of microstructure characteristics with different mixture systems [17]. Combining experimental and simulation methods, Su et al. proposed that the benzene ring has a great influence on the synergistic effect for betaine and anionic surfactants mixture systems [18]. Ergin et al. studied the aggregation behavior of betaine and sodium dodecyl sulfate (SDS) surfactant mixtures at the gas–liquid interface from the perspectives of the polar head, tail chain and water structure through MD simulation [19]. In the previous study, we focused on the change of the configuration entropy of betaine molecules at the interface, bulk phase and very dilute solution [20]. The distribution of local configuration entropy in betaine micelle was used to study the state of the micelle core [21]. In this paper, we focus on the effect of surface adsorption areas of betaine (n-dodecyl betaine, NDB) molecules on its microstructure and properties at the gas–liquid interface.

## 2. Results and Discussion

### 2.1. Distribution of Water Molecules around Polar Heads

**Figure 1:**
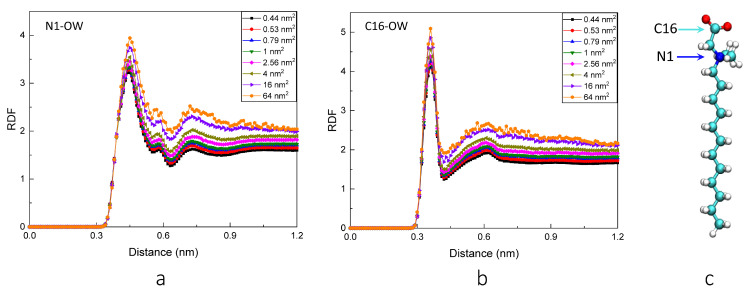
The RDFs of water molecules around N1 and C16 atoms. (**a**,**b**) represent the RDF between N1 and water molecules and the RDF between C16 and water molecules, respectively. (**c**) shows the structure of the NDB molecule and labels the calculated N1 and C16 atoms.

In order to study the distribution of water molecules around the polar heads, we calculate the radial distribution functions (RDFs) of water molecules around the N (N1) and carboxyl-C (C16) atoms of polar heads. As shown in Figure 1, the curve shapes and the locations of peaks hardly change with the increase in surface area. However, as the interfacial area per surfactant increases from 0.44 to 64 nm^2^, the heights of different peaks gradually increase. This means that the local densities of water molecules around peak locations increase, which can also be confirmed by the analysis of the number of hydrogen bonds in the later section. The first peaks of all RDFs around the N1 and C16 atoms are around 0.44 nm and 0.35 nm, respectively. This difference is reasonable because the CH_2_ and CH_3_ groups are connected with the N atom to cause some steric hindrance, and the C16 atom has closer contact with the water phase than the N atom in the view of the NDB structure. The location of the first peak for C16-OW RDF indicates the existence of hydrogen bonds between carboxyl groups and water molecules. From Figure 1, both the N1-OW and C16-OW RDF curves have two peaks. The difference is that there is an acromion on the right side of the first peak for N1 atom, which is located at about 0.58 nm. One interesting finding is that the location of the first peak for N1-OW RDF is the one of the first valley for C16-OW RDF, and the location of the second peak for N-OW RDF is the one of the second valley for C16-OW RDF.

**Figure 2:**
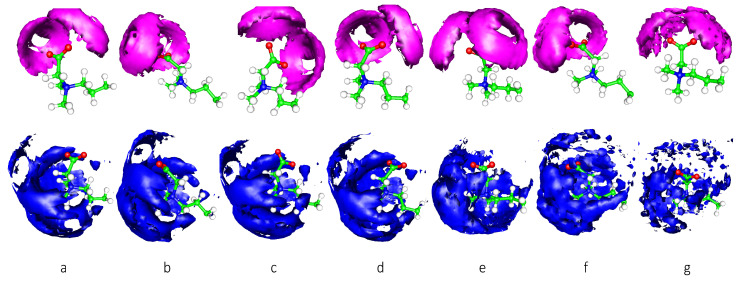
The SDFs of water molecules around C16 and N1 atoms. The top seven graphs present those between C16 and water molecules, and the bottom seven graphs present those between N1 and water molecules. The (**a**–**g**) symbols represent 0.44 nm^2^, 0.53 nm^2^, 0.79 nm^2^, 1 nm^2^, 2.56 nm^2^, 4 nm^2^, 16 nm^2^, and 64 nm^2^ systems, respectively. In order to maintain clarity, only a portion of the tail chain is displayed.

RDF analysis provides the averaged distribution of other atoms or molecules around the specifically functional group, while the spatial distribution function (SDF) provides a more detailed three-dimensional distribution. The SDFs of the first shell water molecules around the N1 and C16 atoms for all the studied systems are plotted in Figure 2. The graph shows the similar distributions around all polar heads, specifically around the oxygen (O) atom on the outer side of the C=O bond on the polar head. For the carboxyl group, water molecules are mainly located around two O atoms, presenting a circular distribution. There is basically no water distribution in the direction of the extension line of the C=O bond. Water molecules exhibit a relatively broad distribution around nitrogen (N) atoms. As the interfacial area per surfactant increases from 0.44 to 64 nm^2^, the distributions of water molecules around the N1 and C16 atoms become more fragmented, especially for those around N1 atoms. This change indicates a higher level of freedom for the surrounding water molecules. It should be noted that the N1 atom already has four covalent bonds, which prevents the N1 atom from forming hydrogen bonds with water molecules. The surrounding water molecules are restrained by weak interactions such as van der Waals forces.

### 2.2. Hydrogen Bonds between Water Molecules and Polar Heads

The hydrogen bond is a type of intermolecularly chemical bond, which typically refers to the interactions between the hydrogen atom and the more electronegative atom (such as nitrogen, oxygen, fluorine) in another molecule. The hydrogen bond is often stronger than other non-covalent bonds and can have important effects on many biochemical and physical processes. The hydrogen bonds are determined using a geometric criterion, where the distance between the chosen donor–acceptor pairs is within 3.5 Å and the H-O-H angle is less than 120°. Based on this definition, the hydrogen bond numbers between water molecules and polar heads are calculated. Table 1 shows the averaged hydrogen bond number of one polar head. From the table, the number of hydrogen bonds increases from 4.14 to 4.98 as the interfacial area per surfactant increases from 0.44 to 64 nm^2^. At a small surface area per surfactant, the interfacial concentration is large, and surfactant molecules tend to aggregate at the interface. Thus, some water molecules around the interface are displaced, resulting in a small decrease for the hydrogen bond numbers. To give more detailed information for the water molecules around polar heads, the coordination number is calculated. The coordination number is calculated by integrating RDFs from zero to the first location of the valley, which counts the coordination numbers of water molecules in the first hydration layer. Table 1 lists all the counted coordination numbers. As the interfacial area per surfactant decreases from 64 to 0.44 nm^2^, the coordination number also decreases, which is consistent with previous discussions of the hydrogen bonds between water molecules and polar heads. Differently, all coordination numbers of the same interfacial area per surfactant are larger than the hydrogen bond numbers, meaning that not all coordinated water molecules around the polar heads can form hydrogen bonds with polar heads. This phenomenon is reasonable because the hydrogen bonds are defined with rigorously geometric criteria. Some water molecules are restrained around the polar heads but cannot satisfy the criteria to form hydrogen bonds with the polar heads.

**Figure 3:**
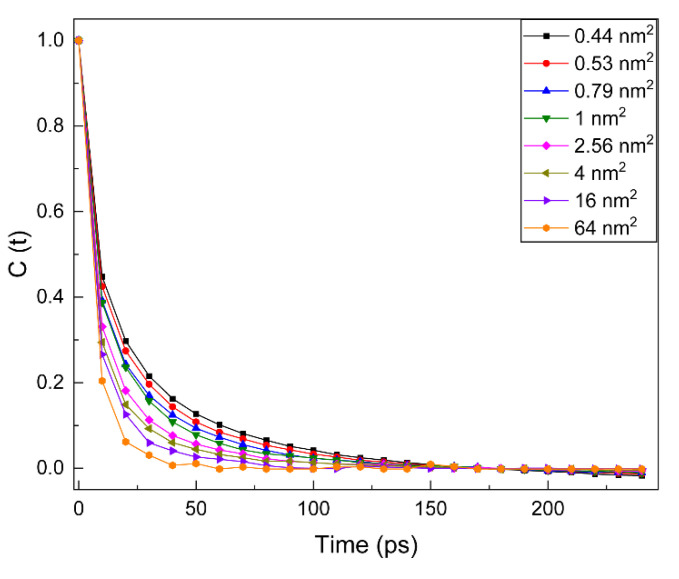
Time correlation function C(t) for the hydrogen bonds formed between polar heads and water molecules.

To further study the interactions between water molecules and polar heads, we calculate the time correlation function C(t) of hydrogen bonds for all systems as shown in Figure 3. With the increase in surface area, the relaxation curves decline more rapidly. This indicates that the water molecules around the NDB head group in systems with smaller surface concentrations are freer to move, and the formed hydrogen bonds are weaker. In addition, the larger the surface concentration of NDB systems, the slower their relaxation curves decline. This suggests that the water molecules around the polar heads in systems with larger surface concentrations are more restricted and less likely to escape. To verify these phenomena, the lifetimes of hydrogen bonds are calculated by integrating the C(t), which is fit through the multiple exponential function of $C\left(t\right)=\sum a_{i}e^{\left(-t/b_{i}\right)}$ [22,23]. As shown in Table 1, the lifetime of hydrogen bonds decreases from 14.84 to 8.10 ps with the increase in surface area from 0.44 to 64 nm^2^. This decrease is consistent with the analysis of C(t) curves in different surface coverages. A longer lifetime indicates the stronger hydrogen bond interactions at large surface coverage.

### 2.3. Tilted Angle at the Interface

**Figure 4:**
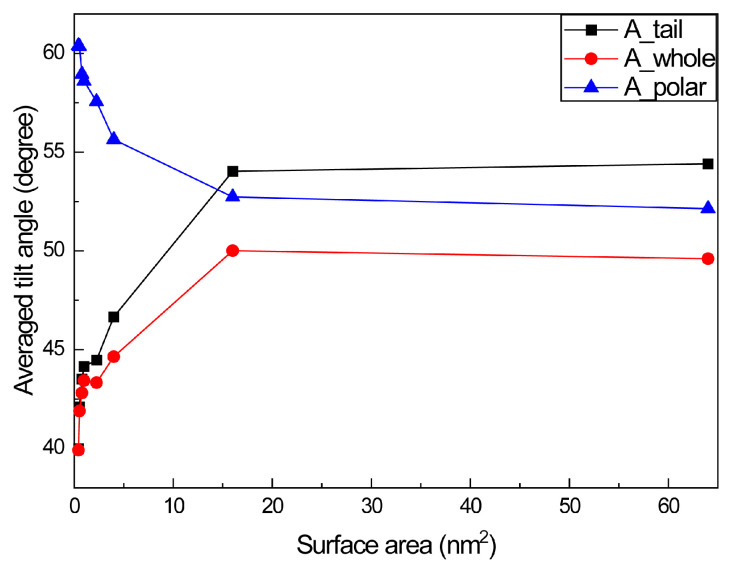
Tilted angles of polar head, tail chain and whole NDB molecule at different surface area.

A very important analysis index of surfactant adsorption is the tilted angle of the surfactant molecule at the air–water interface. In this study, we choose several types of tilted angles along the *Z*-axis (surface normal) to analyze. As shown in Figure 4, the tilted angle between the tail chain and Z-direction (A_Tail), tilted angle between the whole NDB molecule and the Z-direction (A_Whole), and the tilted angle between the polar head and the Z-direction (A_Polar) are considered. All calculated results are averaged. From Figure 4, A_Tail is defined by the angle between the C_4_→C_15_ vector and Z-direction away from the water phase, A_Whole is defined by the angle between the vector C_16_→C_15_ and Z-direction away from the water phase, and A_Polar is defined by the angle between the vector C_16_→C_4_ and Z-direction away from the water phase. Through these definitions, the 0° and 180° angles mean the perpendicular orientation of selected vectors to the vapor–water interface. Differently, the 0° angle denotes the preference to vapor phase, while the 180° angle denotes the preference to water phase. The 90° angle represents that the selected vector is parallel to the vapor–water interface. When 90° is reached, the NDB molecule or tail chain or polar head lies on the interface.

From Figure 4, we can see that A_Tail and A_Whole increase as away from the water phase surface area increases. This is reasonable because fewer NDB molecules are absorbed at the vapor–water interface at a large surface area. With the increasement of surface area, a larger extending space among NDB molecules exists and the preference of away from the water phase NDB molecule and alkyl chain to the interface also increases. A singly absorbed NDB molecule at the interface (64 nm^2^) can have the largest extending space, making whole molecule and tail chain incline to the interface. In comparison, the angle change is small (<5°) when the surface area is small. This is similar to experimental results that indicate the tilted angle hardly changes with the change of surface concentration [5]. At a very large surface area (corresponding to extremely dilute solution), the angle rises ca. 10°. On the contrary, A_Polar decreases with the increase in surface area, showing an opposite trend with A_Tail and A_Whole. This opposite trend may be related to the more alkyl chain lying on the interface at a large surface area. The deflection of the tail chain will also deflect with the polar head, which makes the angle between the polar head and Z-direction smaller. Since the arrangement of the polar head and tail chain has mutual affect, the angle distribution of the overall molecule is slightly lower than that of the tail chain. In comparison, A_Polar angles are larger than A_Tail and A_Whole angles at small surface area (<16 nm^2^), indicating that the tail chain has a larger tendency to surface normal than the polar head. From 16 nm^2^, the A_Tail angle begins to be larger than the A_Polar angle. When the surface coverage is low, the NDB polar head prefers to surface normal more than the tail chain. The change of surface coverage has different effects on the inclination of NDB polar heads and alkyl chain at the air–water interface.

### 2.4. Gauche Defects of NDB Molecule

**Figure 5:**
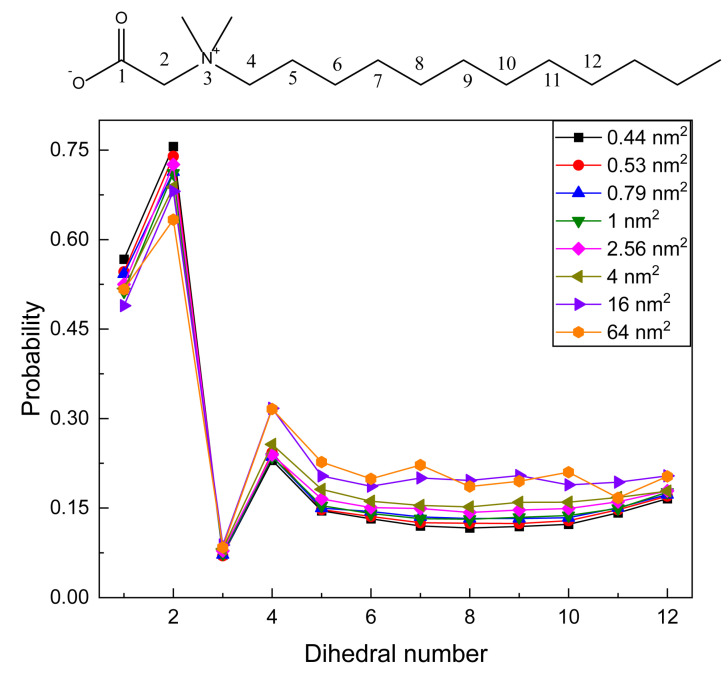
Gauche defects of NDB molecule of different studied systems.

The distribution of dihedral conformations can be well described by the probability of gauche defects. A dihedral angle that deviates from 180° exceeding 60° is defined as a gauche angle. Twelve dihedral angles are defined and calculated. As shown in the mark at the top of Figure 5, mark 1 is defined as a CCNC dihedral angle, and gradually, mark 12 is a CCCC dihedral angle at the end of the tail chain. All curves in Figure 5 have similar distribution trends. From dihedral 1 to 12, the gauche probabilities begin from ca. 0.5 to the highest value at dihedral 2, and the probability rapidly decreases to the lowest value (<0.1) at dihedral 3. For dihedral 4, it again increases. Then, the probability shows a U-shape distribution from dihedral 5 to dihedral 12 at surface areas less than 16 nm^2^. When the surface area is large for 16 and 64 nm^2^, the gauche probability reaches the approximate platform from dihedral 5. Until the last one (dihedral 12), it slightly increases. In detail, the gauche probabilities of dihedral 1 of all systems are around 0.5, which means that gauche and trans conformations account for half, respectively. For dihedral angle 2, gauche defects reach the highest value, showing that the proportion of gauche conformation is high and this chain section bends to a large extent. Dihedrals 1 and 2 are two dihedral angles including the polar head, and their gauche distributions slightly decrease with the increase in surface area. For dihedral 3, gauche defects decrease to about 0.08, which means that the trans conformation occupies a very high proportion. Interestingly, the probability of dihedral 3 hardly changes with the increase in surface area. Contrary to dihedrals related to polar head, the gauche defects of dihedrals 4–12 along the alkyl chain increase with the increase in surface area. For all systems, the gauche probabilities related to the polar head are larger than those with alkyl chains. 

**Figure 6:**
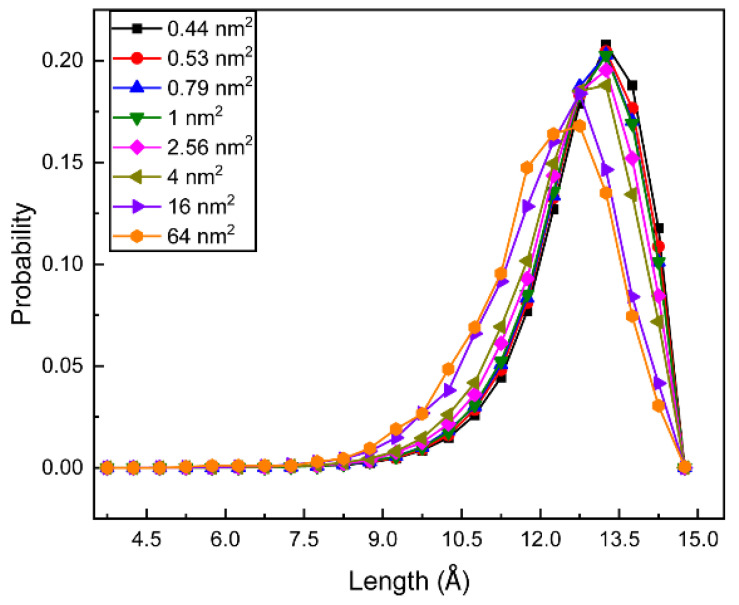
Length distributions of NDB tail chain for different studied systems.

Gauche defects along the tail chain are related to the length of the alkyl chain. The higher the gauche defects, the shorter the chain length. The length distribution of the NDB chain is shown in Figure 6. With the increase in surface area, the distribution peak shifts to the decreasing direction of the tail length. This trend is in good agreement with the one of chain gauche distribution. When the NDB molecule has a small surface area, the interfacial monolayer is compact. The whole NDB molecule and alkyl chain have less of a chance of spreading on the air–water interface and will extend more uprightly, causing more trans-conformations and a longer alkyl chain.

### 2.5. The Thickness of Different NDB Monolayers

The thickness of the interfacial monolayer can be calculated with the “10–90” rule [24,25]. The length between 10% and 90% of the bulk value on the number density profiles of water is defined as the thickness of the interfacial monolayer. The calculated monolayer thicknesses are listed in Table 2. As the interfacial area increases, the thickness decreases and eventually reaches around 0.6 nm. This phenomenon is caused by two factors discussed earlier: firstly, in extremely dilute conditions, alkyl chains bend more severely with more gauche defects and become shorter; secondly, in extremely dilute conditions, the tilt of the NDB molecule is the largest, and the NDB molecule is more likely to be parallel to the interface rather than perpendicular.

### 2.6. Weak Interaction Analysis

**Figure 7:**
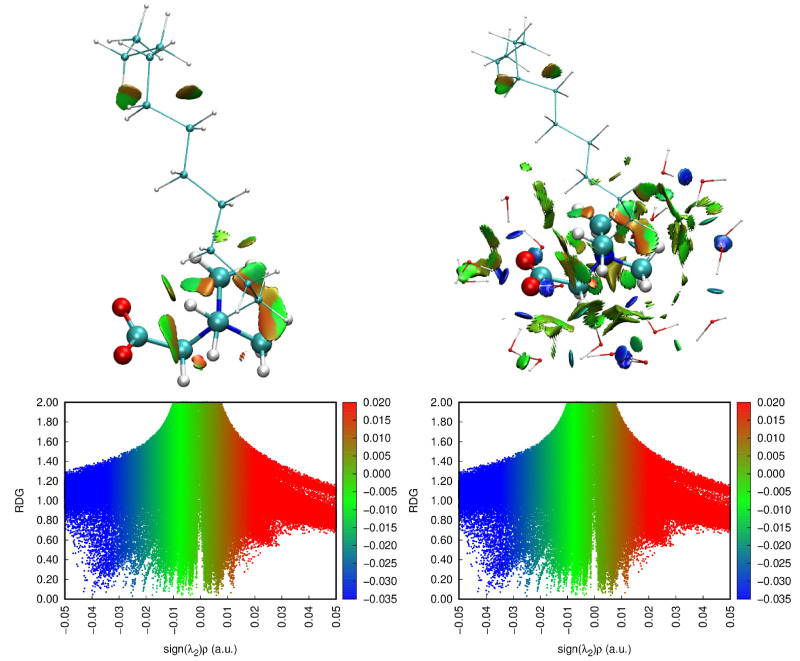
The reduced density gradient (RDG) vs. sign(λ_2_) × ρ(r) for NDB and water molecules. The red, green, and blue colors in the color bar represent the repulsion, van der Waals, and attractive interactions, respectively.

The interactions between different components of the NDB molecule and the interactions between NDB and water molecules are analyzed with the method [26] proposed by Johnson et al. The reduced density gradient function (RDG) is defined as $RDG\left(r\right)=\frac{1}{2(3\pi^{2}{)}^{1/3}}\frac{\left|\nabla \rho \left(r\right)\right|}{\rho (r{)}^{4/3}}$ [27]. As shown in Figure 7, red, green, and blue colors in the color bar represent the repulsion, van der Waals, and attractive interactions, respectively. The sign of *λ*_2_ is utilized to distinguish bonded (*λ*_2_ < 0) and nonbonded (*λ*_2_ > 0) interactions. The quantity sign(*λ*_2_)ρ represents the product of ρ and the sign of *λ*_2_, where *λ*_2_ is the second largest eigenvalue of the Hessian matrix of ρ. One or more spikes that appear in low-density and low-gradient regions indicate the existence of weak interactions. Figure 7 is generated with the Multiwfn 3.8 program [28] and VMD 1.9.3 [29].

For one single molecule, the weak interaction isosurface will appear in the area where the dihedral angle bends more severely. From Figure 7, green and red parts in the isosurface represent van der Waals interactions and spatially potential resistance interactions, respectively. The gauche defects of the dihedral angles on the polar head are higher; thus, green and red fragments are obvious near the polar head. Furthermore, some green and red distributions exist at terminal tail parts, which also correspond to the increased distribution of gauche defects. In order to study the interactions between NDB and water molecules, we select all water molecules in the first hydration layer around NDB molecules to calculate. In regions around polar heads, more green and red parts occur for NDB and the water system. Moreover, some blue fragments appear, which correspond to the hydrogen bonds formed between O atoms of the polar head and water molecules or between water and water molecules around the polar head. It should be noted that N atoms cannot form hydrogen bonds with water molecules due to the four bonds connected by N atoms.

## 3. Materials and Methods

The optimized NDB structure was obtained by Gaussian 09 software. All MD simulations were performed using GROMACS 2019 software [30]. The general AMBER force field (gaff2) is used as the force field. The atom-centered point charges are obtained with the restrained electrostatic potential (RESP) methods [31] at HF/6-31G* level. TIP3P water models [32] were applied. To build initial models, the 8 × 8 × 24 nm^3^ rectangular box with the *Z*-axis being perpendicular to the interface was used. In the center of the box, a water slab of 8 × 8 × 8 nm^3^ was placed to construct the water–vapor interface with the *gmx editconf* command. Equal amounts of NDB molecules were respectively placed at two sides of the water slab to build surfactant layers with the *gmx solvate* command. All trans alkyl chains of NDB molecules extended away from the water phase and were perpendicular to the interface. For each system, corresponding NDB molecules were added to obtain interfacial systems from extremely dilute surface concentration to saturated concentration. Detailed information of all simulated systems is provided in Table 3. In the table, the change of surface concentration was characterized by the change of surface adsorption area of one NDB molecule. For example, for a system with an area of 64 nm^2^, one molecule was placed on each of two interfaces, resulting in a total of two NDB molecules in the system. The surface area of each interface is 8 × 8 nm, which amounts to 64 nm^2^; hence, it is labeled as 64 nm^2^. Other systems are labeled similarly. It should be noted that the decreasing interface area occupied by each molecule from 64 to 0.44 nm^2^ means that more NDB molecules are gathering together. After constructing initial models, the steepest descent method was applied to minimize systems. Then, the NVT simulation with 200 ns time-length was carried out to equilibrate the system and obtain MD trajectories. To keep the temperature of 298 K, the v-rescale temperature coupling method was used [33]. During the simulation, the LINCS algorithm was used to constrain the bond length [34]. To calculate electrostatic interactions, the particle mesh Ewald (PME) summation method was used [35]. The 1.0 nm cut-off length was set for van der Waals interactions. 

## 4. Conclusions

**Figure 8:**
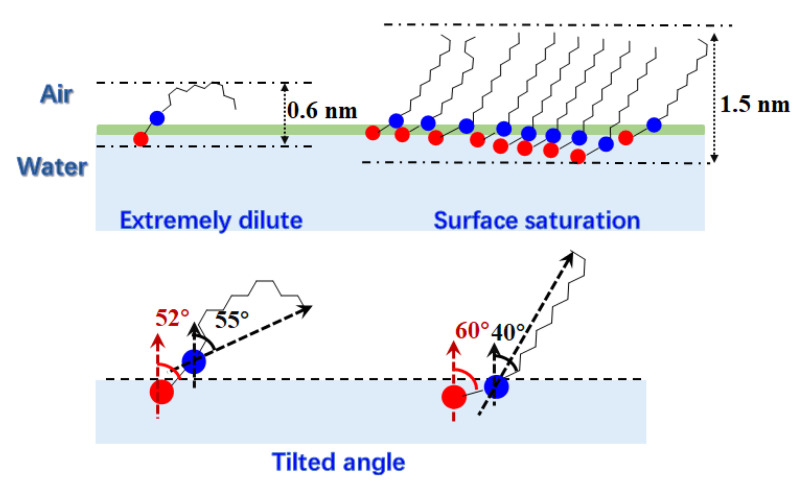
The schematic diagram of typical NDB monolayers at saturation adsorption and extremely dilute solution. Red and blue balls in the figure represent the negative and positive charge center, respectively.

At the air–water interface, the structures and properties of NDB monolayers from extremely dilute surface coverage to saturated surface coverage are analyzed with MD simulations. Interactions between NDB monolayers and water molecules are analyzed by the RDF, SDF and hydrogen bond analyses. As the surface area increases, the RDF locations and SDF graphs are similar. For the polar carboxyl group, water molecules are mainly located around oxygen atoms with two circular distributions. Although the number of hydrogen bonds between polar heads and water molecules and coordination numbers of water molecules around polar heads increase with the increase in surface area, the time correlation function decays faster, and the lifetime of hydrogen bonds decreases. Hydrogen bonds formed at small surface areas are stronger than those at large surface areas. An analysis of weak interactions also shows the hydrogen bonds’ interactions between polar heads and water molecules. As more NDB molecules are adsorbed at the surface, gauche defects including the polar head increase, but gauche defects along the alky chain decrease. The NDB alkyl chains become longer at large surface coverage. Moreover, gauche defects related to the polar head are larger than those with alkyl chains for each NDB monolayer, which is consistent with the results of the weak interaction iso-surface. Both the tail chain and NDB molecule tilt further away from the surface angle with the increase in surface area, while the polar head shows the opposite change. When surface coverages are large, the change of tilted angle is little. The tail chain and whole molecules rises ca. 10°, but the polar head reduces ca. 7° under extremely dilute conditions. Contrary to intuitive thoughts, the tilted angle shows that polar heads are more inclined toward the interfacial plane at large surface coverages, while tail chains prefer to tilt toward the interface at low surface coverages. Based on the MD results, typical NDB monolayers at saturation adsorption and extremely dilute condition are proposed as shown in Figure 8. The MD models of NDB monolayers give a molecule-level presentation of different arrangements for NDB molecules at the air–water interface, monolayer thickness, and the inclination of NDB fragments at the air–water interface. This study provides the theoretical reference for further exploration and application of NDB adsorption at the interface and for other zwitterionic surfactant micelle systems.

## Figures and Tables

**Table 1:** The hydrogen bond numbers, coordination numbers and lifetimes between water molecules and polar heads.

System	0.44 nm^2^	0.53 nm^2^	0.79 nm^2^	1 nm^2^	2.56 nm^2^	4 nm^2^	16 nm^2^	64 nm^2^
Hydrogen bond number	4.14 ± 0.08	4.21 ± 0.09	4.31 ± 0.11	4.36 ± 0.13	4.47 ± 0.19	4.61 ± 0.24	4.85 ± 0.45	4.98 ± 0.87
Coordination number	5.31 ± 0.11	5.47 ± 0.13	5.62 ± 0.16	5.70 ± 0.19	5.89 ± 0.29	6.12 ± 0.36	6.60 ± 0.64	6.89 ± 1.21
Lifetime	14.84 ps	14.17 ps	12.94 ps	12.31 ps	10.78 ps	9.75 ps	8.63 ps	8.10 ps

**Table 2:** The interfacial thickness of different NDB monolayers.

			Interfacial	Thickness	(nm)			
System	0.44 nm^2^	0.53 nm^2^	0.79 nm^2^	1 nm^2^	2.25 nm^2^	4 nm^2^	16 nm^2^	64 nm^2^
Thickness	1.50	1.48	1.40	1.33	0.97	0.74	0.62	0.60

**Table 3:** Detailed composition of studied systems.

	0.44 nm^2^	0.53 nm^2^	0.79 nm^2^	1 nm^2^	2.56 nm^2^	4 nm^2^	16 nm^2^	64 nm^2^
NDB	288	242	162	128	50	32	8	2
H_2_O	16,586	16,667	16,824	16,904	17,042	17,074	17,115	17,128

## Data Availability

The data presented will be made available on request by the corresponding authors.
